# Drug-coated balloon treatment for TASC C/D infrapopliteal disease: Two-year matched cohort outcomes

**DOI:** 10.17305/bb.2025.12157

**Published:** 2025-06-10

**Authors:** Yu Yan, Haixia Tu, Mingxuan Li

**Affiliations:** 1 Department of Vascular Surgery, Beijing Fengtai You’anmen Hospital, Beijing, China; 2 Department of General Surgery, Beijing Daxing District Hospital of Integrated Chinese and Western Medicine, Beijing, China

**Keywords:** Infrapopliteal arterial disease, IPAD, drug-coated balloon, DCB, primary patency, PP, target lesion revascularization, TLR

## Abstract

As the most common form of peripheral arterial disease, lower extremity arterial disease—caused by atherosclerotic stenosis or occlusion—has led to widespread concern due to the high risk of postoperative restenosis. This study aimed to evaluate the effectiveness of drug-coated balloon (DCB) angioplasty in treating severe infrapopliteal artery (IPA) lesions. Plain old balloon (POB) angioplasty served as the control. Patients who underwent procedures at our center for Trans-Atlantic Inter-Society Consensus (TASC) C/D IPA lesions between June 2020 and June 2022 and met the inclusion criteria were enrolled in this retrospective cohort study, which used the propensity score matching (PSM) method. The primary outcomes were the 2-year cumulative rates and survival trends of primary patency (PP) and target lesion revascularization (TLR), based on the treated lesions. Secondary outcomes included limb-based major amputation (MA) and patient-based all-cause death (ACD). A total of 278 target lesions were initially included, with significant differences (*P* < 0.05) observed in some non-outcome variables. After PSM, analyses were conducted on 240 target lesions, 221 limbs, and 195 patients. The PSM models satisfied both the common support and parallel trend assumptions. In terms of PP, the 2-year cumulative rate in the DCB group was significantly higher than in the POB group (48.0% vs 22.9%, *P* < 0.001). The log-rank test yielded a *P* value of < 0.001, and the adjusted hazard ratio (HR) from Cox regression analysis was 2.303 [95% confidence interval (CI): 1.518–3.495]. However, there was no statistically significant difference in TLR between the two groups: the 2-year cumulative rates were 25.0% vs 27.1% (*P* ═ 0.767), the log-rank test *P* value was 0.563, and the adjusted HR was 0.956 (95% CI: 0.523–1.747). Similarly, no significant differences were found between groups in MA or ACD (*P* > 0.05). Based on these findings, the study concludes that for severe IPA lesions such as TASC C/D, DCB angioplasty is superior to POB angioplasty in maintaining PP over a 2-year period, without any inferiority in other clinical outcomes.

## Introduction

Infrapopliteal arterial disease (IPAD), with or without femoropopliteal inflow disease, is the primary cause of critical limb ischemia (CLI) [1], [2]. Femoropopliteal-to-distal bypass surgery is considered the traditional treatment option for revascularization in IPADs [3–5]. In the past few decades, percutaneous transluminal angioplasty (PTA) [i.e., plain old balloon angioplasty (POBA) alone], which is more minimally invasive, has been widely used, especially for patients whose physical conditions make it difficult to withstand open surgery or who do not have suitable distal arteries for bypass [6–10]. However, although this modality has a satisfactory technical success rate, it still has a significantly high risk of clinical failure caused by lesion restenosis even in the short term [11–13].

The superiority of drug-coated balloon (DCB) angioplasty for femoropopliteal artery lesions over POBA has been demonstrated [14–16] in recent years. However, the exploration path for the superiority of DCBs in the treatment of infrapopliteal artery (IPA) lesions is relatively tortuous. Compared with POBA alone, significantly lower cumulative rates of target lesion restenosis and revascularization at the 1st year after DCB angioplasty (DCBA) have been reported by Schmidt et al. and Liistro et al., respectively [17], [18]. However, in the later IN. PACT Deep trial [19], which included 358 patients, the cumulative rate of target lesion revascularization (TLR) at the 1st postoperative year was not significantly different between the groups (11.9% vs 13.5%, *P* ═ 0.682). Moreover, in the BIOLUX P-II trial [20], there were no statistically significant differences in the cumulative rates of the following outcomes during the same follow-up period (*P* > 0.05): TLR (30.1% vs 30.6%), patency loss (50.8% vs 45.6%), and major amputation (MA, 3.3% vs 5.6%). However, the conclusion of the AcoArt II-BTK trial from China published in 2021 favored DCB [21]. This study included 79% chronic total occlusion (CTO) lesions and reported better results for the DCB group with 6-month primary patency (PP, 75.0% vs 28.3%, *P* < 0.001) and 1-year TLR (8.5% vs 23.2%, *P* ═ 0.028). The Lutonix BTK trial [22], which involved a single arm and included 69.3% of Trans-Atlantic Inter-Society Consensus (TASC) C/D IPA lesions [5] (Figure S1), reported satisfactory DCBA results. However, there are no published controlled studies including only TASC C/D lesions. Thus, this study was conducted in accordance with the STROBE reporting checklist to determine the superiority of DCBs in these severe lesions.

## Materials and methods

### Study design and setting

This study was a single-center retrospective cohort analysis conducted at a comprehensive tertiary hospital in Beijing, China. We searched the hospital’s electronic medical record management system for patients who underwent endovascular therapies for lower extremity arteries from June 2020 to June 2022. All patients who received either DCBA or POBA for TASC C/D IPA lesions and did not meet the exclusion criteria were included in the study. The exclusion criteria were: (1) planned amputation prior to intervention and (2) lack of follow-up data due to loss to follow-up or other reasons. A DCB was defined as any dilatation balloon coated with antiproliferative drugs (such as paclitaxel, sirolimus, or everolimus) on its outer surface. The records were divided into two independent groups based on the intervention modalities: the DCB group and the control group (POBA). A single patient could not be included in both groups simultaneously, and preference was given to inclusion in the DCB group. Data extraction and analysis were subsequently performed. Case screening was conducted independently by YY and HT, with any disagreements resolved by ML after discussion.

### General treatment procedure

In this retrospective study, although we did not require the processes experienced by the included cases to be consistent with our established general treatment procedure, this procedure has been widely followed. The main criteria included the following: (1) the patient received preoperative antiplatelet therapy for more than one month; if not, a loading dose of the drug (aspirin or clopidogrel, 300 mg) was administered on the day of the operation; (2) the patient continued antiplatelet therapy for at least six months postoperatively; (3) skin puncture points for the endovascular operation could be located on either the ipsilateral or contralateral side; (4) intraoperatively, a guide wire system was established through the target lesion in the true lumen, and a balloon was placed along the guide wire for dilatation; (5) the diameter of the balloon did not exceed 120% of the diameter of the reference vessel; (6) after DCBA, POBA at the same location was no longer performed; (7) the patient was asked to attend follow-up visits at one, three, six, twelve, eighteen, and twenty-four months postoperatively unless they visited voluntarily due to complaints; and (8) at each follow-up, a condition inquiry, physical examination, ankle-brachial index (ABI) assessment, and imaging studies (i.e., Doppler ultrasound [DUS], computed tomography angiography [CTA], or quantitative vascular angiography [QVA]) were conducted, followed by an evaluation of outcomes of interest and Rutherford’s classification (RC).

Additionally, if restenosis of the lumen diameter of a treated lesion is found to reach 50% after the operation, early intensive drug intervention will be initiated regardless of whether symptoms of lower limb ischemia reappear. This intervention should include at least the following: increasing the dosage of antiplatelet drugs and/or adding anticoagulant drugs, increasing vasodilators such as prostaglandins, and instructing patients to enhance lower limb exercise, such as brisk walking. Regardless of whether restenosis is alleviated after the aforementioned drug intervention, TLR will only be considered if symptoms reappear and reach at least RC-3. Notably, some patients refused to undergo revascularization.

### Variables and data

When data were extracted, all observations focused on the target lesion, defined as an IPA lesion classified as TASC C/D that had undergone the corresponding intervention modality (i.e., DCBA or POBA) for its group. An arterial site with stenosis of less than 30% of the diameter of the nearby reference artery was considered a “normal site.” Lesions separated by a normal site with a distance of less than 20 mm or those that underwent the same balloon dilatation simultaneously were considered part of the same target lesion in total length; otherwise, they were deemed separate and distinct target lesions. The variables were broadly categorized into five periods: preoperative demographics, angiographic findings before intervention, intraoperative intervention, short-term postoperative medication and complications, and follow-up. All variables with potentially different definitions (such as calcification and dissection classification [23, 24]) were evaluated according to pre-established unified criteria. The inclusion period for postoperative follow-up data for all patients was 2 years (690–750 days). To avoid bias due to abnormalities [25], if the preoperative ABI of an affected limb was ≥ 1.4, the postoperative ABI was recorded as a missing value.

**Figure 1.**
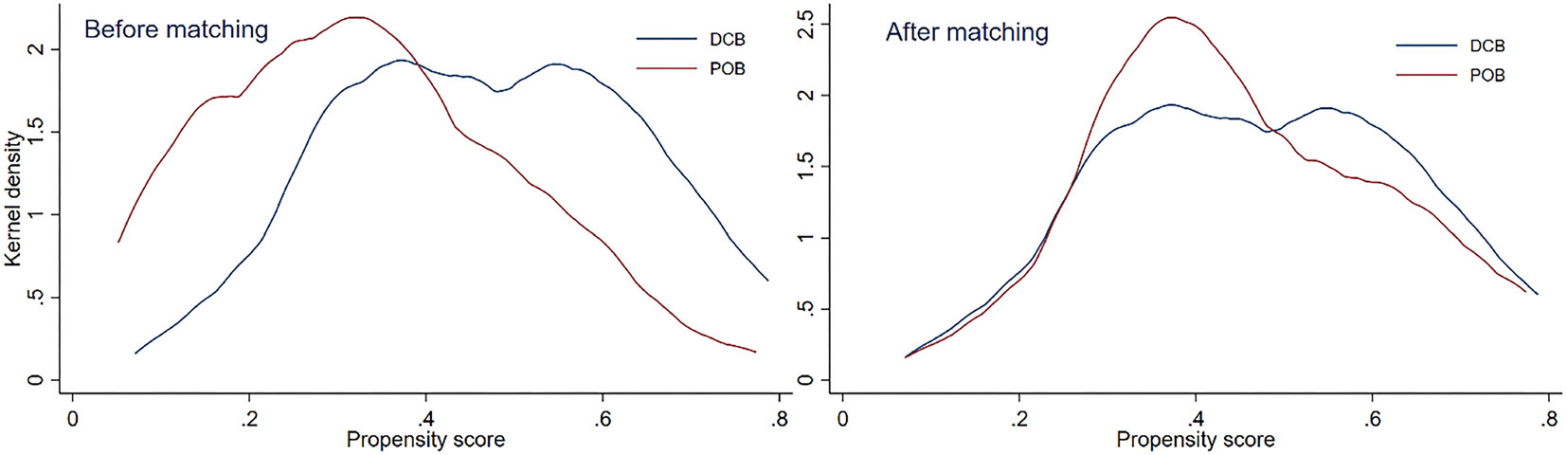
**Comparison of the kernel density distribution before and after propensity score matching for the data based on target lesion.** DCB: Drug-coated balloon; POB: Plain old balloon.

We considered the primary outcomes to be the PP and TLR (both based on the target lesion) and the secondary outcomes to be MA (based on the limb) and all-cause death (ACD, based on the patient). The PP was defined as freedom from restenosis (defined as <50% residual lumen diameter under CTA/QVA or peak systolic velocity ratio ≥2.4 under DUS) without TLR. All DUS examinations were performed by experienced vascular ultrasound professionals. TLR was defined as repeat percutaneous or surgical intervention due to angiographic evidence of >50% restenosis, accompanied by recurrence of pain in the foot and/or the presence of a nonhealing limb ulcer or gangrene. MA was defined as amputation above the ankle. The outcome measures included the cumulative rates of the above outcomes at the follow-up endpoint, as well as the hazard ratios (HRs, DCBA vs POBA) with their 95% confidence intervals (CIs).

After data extraction, dataset No. 0 was obtained, which was based on the units (i.e., target lesion, limb, or patient) corresponding to their original meanings. To standardize the units for each analysis, data merging was performed. The specific methods included: taking the most severe value (such as the RC), taking the mean value (such as the length of the target lesion), and accounting for missing values (such as the location of the target lesion). The following datasets, based on the original units for each outcome of interest, were subsequently obtained: No. 1, based on the target lesion (PP and TLR); No. 2, based on the limb (MA); and No. 3, based on the patient (ACD).

**Table 1:** Preoperative data based on target lesion in the analyses of primary patency and target lesion revascularization

**Variable**	**Before propensity score matching**			**After propensity score matching**		
	**DCB group (*n* ═ 107)**	**POB group (*n* ═ 171)**	***P* value**	**DCB group (*n* ═ 100)**	**POB group (*n* ═ 140)**	***P* value**
Duplicate patient	25 (23.4)	32 (18.7)	0.363	23 (23.0)	24 (17.1)	0.322
Duplicate limb	13 (12.2)	12 (7.0)	0.195	12 (12.0)	11 (7.9)	0.374
Duplicate artery	4 (3.7)	3 (1.8)	0.435	4 (4.0)	3 (2.1)	0.455
Restenosis	20 (18.7)	31 (18.1)	1.000	20 (20.0)	26 (18.6)	0.868
Previous revascularization	27 (25.2)	37 (21.6)	0.558	26 (26.0)	30 (21.4)	0.441
Left side	58 (54.2)	93 (54.4)	1.000	52 (52.0)	81 (57.9)	0.430
Preoperative RC			0.574			0.896
3	5 (4.7)	3 (1.8)		3 (3.0)	3 (2.1)	
4	33 (30.8)	52 (30.4)		32 (32.0)	41 (29.3)	
5	45 (42.1)	77 (45.0)		44 (44.0)	63 (45.0)	
6	24 (22.4)	39 (22.8)		21 (21.0)	33 (23.6)	
Preoperative ABI^†^	0.40±0.13	0.37±0.14	0.078	0.40±0.13	0.37±0.14	0.099
Preoperative ABI exceeding 1.4	8 (7.5)	6 (3.5)	0.164	8 (8.0)	5 (3.6)	0.156
Age (year)	73.3±7.8	71.8±7.6	0.113	73.4±7.8	72.4±7.4	0.337
Male	70 (65.4)	104 (60.8)	0.448	65 (65.0)	88 (62.9)	0.786
Body mass index	25.3±2.0	24.4±2.1	0.002^*^	25.1±2.0	24.6±2.1	0.071
Hypertension	95 (88.8)	123 (71.9)	0.001^*^	88 (88.0)	109 (77.9)	0.060
Diabetes mellitus	93 (86.9)	159 (93.0)	0.137	88 (88.0)	130 (92.9)	0.257
Dyslipidemia	59 (55.1)	67 (39.2)	0.013^*^	52 (52.0)	60 (42.9)	0.190
Coronary heart disease	63 (58.9)	107 (62.6)	0.613	57 (57.0)	97 (69.3)	0.057
Auricular fibrillation	19 (17.8)	55 (32.2)	0.008^*^	19 (19.0)	37 (26.4)	0.216
Chronic kidney disease	24 (22.4)	44 (25.7)	0.569	22 (22.0)	28 (27.1)	0.450
Chronic lung disease	14 (13.1)	32 (18.7)	0.248	12 (12.0)	26 (18.6)	0.210
Anemia	50 (46.7)	76 (44.4)	0.712	49 (49.0)	61 (43.6)	0.432
Previous cerebral infarction	40 (37.4)	46 (26.9)	0.083	36 (36.0)	44 (31.4)	0.489
Previous myocardial infarction	18 (16.8)	36 (21.0)	0.438	18 (18.0)	30 (21.4)	0.624
Current smoking	49 (45.8)	55 (32.2)	0.030^*^	46 (46.0)	49 (35.0)	0.108
Previous smoking	79 (73.8)	109 (63.7)	0.088	72 (72.0)	91 (65.0)	0.265
Preoperative statin^‡^	53 (49.5)	68 (39.8)	0.136	51 (51.0)	63 (45.0)	0.363
Preoperative antiplatelet^‡^	58 (54.2)	83 (48.5)	0.389	54 (54.0)	73 (52.1)	0.794

Observations are presented as “*n* (%)” or “± standard deviation”. ^†^99/165 and 92/135 observations respectively; ^‡^Lasted at least 6 months; ^*^Significant statistical difference due to a *P* value of less than 0.05. RC: Rutherford classification; ABI: Ankle brachial index; DCB: Drug-coated balloon; POB: Plain old balloon.

### Error control

The propensity score matching (PSM) method was employed to filter all included observations and reduce selection bias [26]. To assess statistical power, the sample size derived from PSM was compared with the estimated sample size.

### Ethical statement

The study was conducted in accordance with the Declaration of Helsinki (revised in 2013) and received approval from the institutional ethics committee (No. 2024-03-01). The requirement for individual consent for this retrospective analysis was waived.

### Statistical analysis

The data distribution for numerical variables was represented as “mean ± standard deviation,” while categorical variables were represented as “number (percentage).” Statistical analyses were conducted using Stata (Stata Corp., College Station, TX, United States) version 16.0. All hypothesis tests were two-sided, with a significance level set at 0.05. Univariate analyses for variables other than the outcomes of interest were performed separately on the four datasets using Student’s *t*-test [27] or Fisher’s exact test [28]. Variables that were significantly different and unaffected by the intervention measures were excluded from further analysis.

Using the selected variables, 1:1 nearest neighbor PSM with logit regression was conducted on datasets No. 1, No. 2, and No. 3, employing calipers with widths of 0.01, allowing for replacements of the observations in the plain old balloon (POB) group. The matched cohorts were incorporated separately into their respective datasets. Kernel density plots before and after matching were created and compared to assess whether the regression model met the common support assumption. After matching, univariate analyses were repeated for each dataset. The results were evaluated for compliance with the parallel test assumption by observing changes in intergroup differences for each variable derived from the univariate analyses conducted before and after matching.

Subsequently, based on the matched datasets, the outcomes of interest were measured using the following methods: comparison of cumulative rates between groups, plotting of the Kaplan–Meier (K–M) survival curve with the log-rank test [29], and multivariate Cox regression analysis [30]. In the regression analyses, preoperative ABI values exceeding 1.4 were treated as missing values. Additionally, sample size estimation was performed using PASS (NCSS Corp., Kaysville, Utah, United States) version 15.0, assuming equal numbers between groups. The 2-year cumulative *P* values of the two groups in this study were used to estimate the minimum sample size required for adequate statistical power.

## Results

### Data before matching

A total of 221 patients who met the selection criteria, with 253 affected limbs and 278 target lesions, were included in the study. All participants were Han Chinese, predominantly male (61.1%), and aged between 52 and 90 years. DCBs used were Litos^®^/Tulip^®^ (Acotec Scientific Corp., Beijing, China), which are compatible with a 0.014/0.018-inch wire guide system and have a surface coated with 3 µg/mm^2^ of paclitaxel. Except for body mass index, hypertension, dyslipidemia, atrial fibrillation, current smoking status, and postoperative statin use, the non-outcome variables did not differ significantly between the groups (*P* > 0.05). Details can be found in Tables S1 and S2.

### Primary outcomes

In dataset No. 1, which focused on the target lesion, no significant differences between groups were found for any variables other than the six previously mentioned before matching. After incorporating these six variables into the PSM model, a new dataset, labeled No. 1, was created. Only a small number of observations were excluded (7 out of 107 and 31 out of 171), which satisfied the common support assumption. The kernel density plot indicated that the values of the two groups largely overlapped, supporting the model’s compliance with the assumption (Figure 1). After matching, there were no significant differences in the non-outcome variables between the groups (*P* > 0.05), consistent with the parallel test assumption. Details are presented in Tables 1 and 2.

**Table 2:** Intra- and postoperative data based on target lesion in the analyses of primary patency and target lesion revascularization

**Variable**	**Before propensity score matching**			**After propensity score matching**		
	**DCB group (*n* ═ 107)**	**POB group (*n* ═ 171)**	***P* value**	**DCB group (*n* ═ 100)**	**POB group (*n* ═ 140)**	***P* value**
Arterial location			0.481			0.661
Anterior tibial	38 (35.5)	61 (35.7)		34 (34.0)	46 (32.9)	
Peroneal	6 (5.6)	12 (7.0)		6 (6.0)	12 (8.6)	
Posterior tibial	34 (31.8)	50 (29.2)		31 (31.0)	42 (30.0)	
Tibiofibular trunk - peroneal	15 (14.0)	15 (8.8)		15 (15.0)	14 (10.0)	
Tibiofibular trunk - posterior tibial	14 (13.1)	33 (19.3)		14 (14.0)	26 (18.6)	
TASC classification			0.324			0.511
C	63 (58.9)	90 (52.6)		58 (58.0)	74 (52.9)	
D	44 (41.1)	81 (47.4)		42 (42.0)	66 (47.1)	
Complete IPA before intervention^†^			0.704			0.684
0	65 (60.8)	108 (63.2)		61 (61.0)	90 (64.3)	
1	42 (39.2)	63 (36.8)		39 (39.0)	50 (35.7)	
Calcification classification			0.607			0.590
0 (no visible calcium)	4 (3.7)	5 (2.9)		4 (4.0)	3 (2.1)	
1 (unilateral calcification <5 cm)	9 (8.4)	13 (7.6)		8 (8.0)	12 (8.6)	
2 (unilateral calcification ≥5 cm)	29 (27.1)	62 (36.3)		27 (27.0)	50 (35.7)	
3 (bilateral calcification <5 cm)	46 (43.0)	66 (38.6)		44 (44.0)	56 (40.0)	
4 (bilateral calcification ≥5 cm)	19 (17.8)	25 (14.6)		17 (17.0)	19 (13.6)	
Chronic total occlusion	53 (49.5)	99 (57.9)	0.176	51 (51.0)	82 (58.6)	0.292
Length (mm)	154.2±37.2	158.1±32.3	0.374	154.2±38.3	157.6±33.7	0.466
Reference vessel diameter (mm)	2.54±0.11	2.53±0.12	0.648	2.54±0.11	2.53±0.12	0.642
Intervention to SPAs	16 (15.0)	22 (12.9)	0.720	15 (15.0)	17 (12.1)	0.566
Maximum balloon diameter (mm)	2.98±0.24	2.94±0.28	0.178	3.00±0.24	2.93±0.27	0.064
Maximum dilatation pressure (atm)	13.20±0.85	13.34±0.70	0.141	13.18±0.87	13.36±0.68	0.067
No. of dilatation	2.79±0.71	2.91±0.62	0.166	2.80±0.71	2.91±0.62	0.186
Dilatation duration (sec)	493.8±131.3	510.0±110.0	0.271	495.1±131.4	511.2±110.5	0.303
Subintimal angioplasty	22 (20.6)	46 (26.9)	0.254	21 (21.0)	40 (28.6)	0.229
Retrograde angioplasty	12 (11.2)	28 (16.4)	0.293	11 (11.0)	25 (17.9)	0.199
Crossover	8 (7.5)	21 (12.3)	0.231	7 (7.0)	18 (12.9)	0.198
Stent implantation	1 (0.9)	0 (0)	0.385	1 (1.0)	0 (0)	0.417
Dissection			0.815			0.618
A (minor radiolucent areas)	12 (11.2)	18 (10.5)		11 (11.0)	13 (9.3)	
B (linear dissection)	8 (7.5)	9 (5.3)		8 (8.0)	9 (6.4)	
C (contrast outside lumen)	4 (3.7)	4 (2.3)		4 (4.0)	3 (2.1)	
D (spiral dissection)	1 (0.9)	1 (0.6)		1 (1.0)	0 (0)	
Complete IPA after intervention^†^			0.339			0.555
1	8 (7.5)	12 (7.0)		8 (8.0)	10 (7.1)	
2	65 (60.8)	90 (52.6)		59 (59.0)	74 (52.9)	
3	34 (31.8)	69 (40.4)		33 (33.0)	56 (40.0)	
Device success	107 (100)	171 (100)	1.000	100 (100.0)	140 (100.0)	1.000
Technical success	105 (98.1)	166 (97.1)	0.711	98 (98.0)	137 (97.9)	1.000
Postoperative statin^‡^	97 (90.6)	141 (82.5)	0.078^*^	90 (90.0)	120 (85.7)	0.429
Postoperative antiplatelet^‡^	100 (93.4)	152 (88.9)	0.290	95 (95.0)	128 (91.4)	0.321
Serious complications	1 (0.9)	1 (0.6)	1.000	1 (1.0)	1 (0.7)	1.000
Minor complication: AKI	9 (8.4)	15 (8.8)	1.000	9 (9.0)	14 (10.0)	0.828
Minor complication: MB	5 (4.7)	3 (1.8)	0.267	3 (3.0)	1 (0.7)	0.311
Minor complication: PRP	3 (2.8)	6 (3.5)	1.000	3 (3.0)	4 (2.9)	1.000

Observations are presented as “*n*(%)” or “± standard deviation”. ^†^Infrapopliteal artery directly reaching foot without stenosis of more than 30%; ^‡^Lasted at least 6 months; ^*^The *P* value in the comparison of observations based on patient was 0.042. TASC: Trans-Atlantic Inter-Society Consensus; IPA: Infrapopliteal artery; SPA: Suprapopliteal artery; AKI: Acute kidney injury; MB: Minor bleeding; PRP: Puncture related problem; DCB: Drug-coated balloon; POB: Plain old balloon.

**Figure 2.**
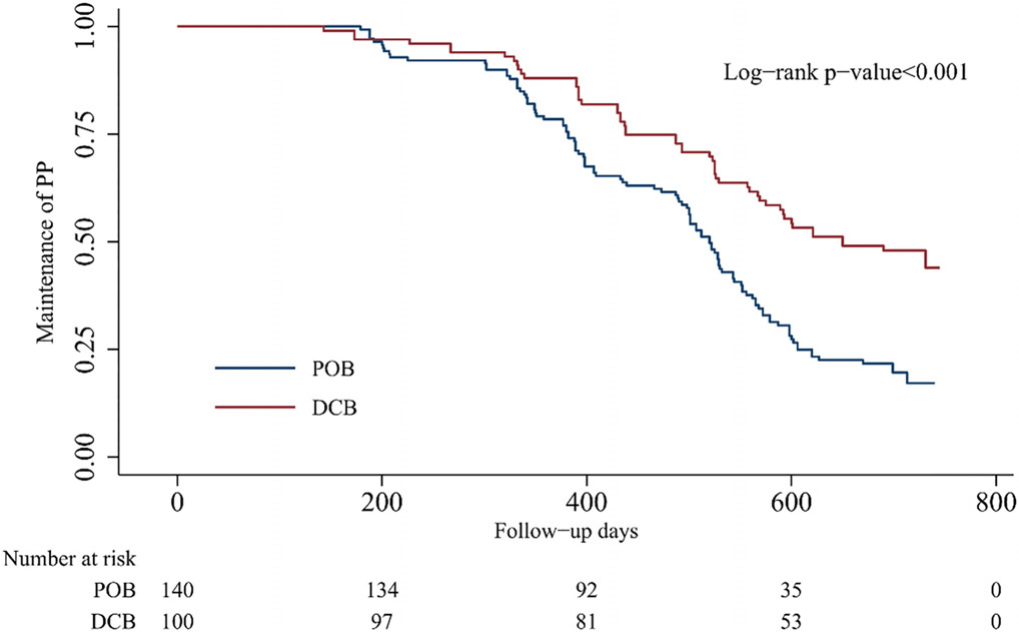
**Kaplan-Meier survival of primary patency.** POB: Plain old balloon; DCB: Drug-coated balloon.

**Figure 3.**
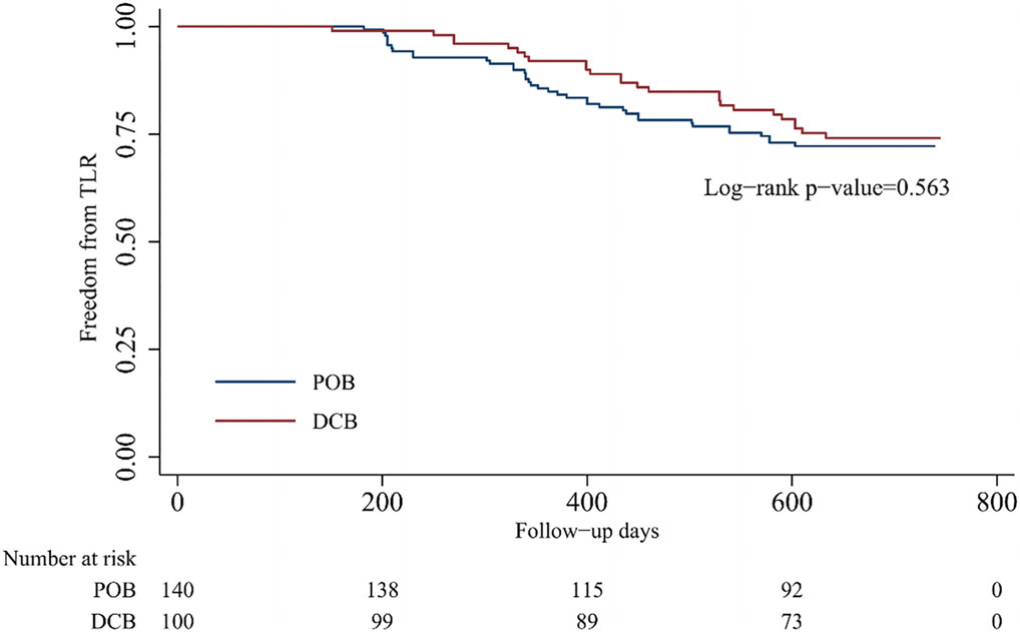
**Kaplan-Meier survival of freedom from target lesion revascularization.** POB: Plain old balloon; DCB: Drug-coated balloon.

A comparison of the matched data showed that the 2-year cumulative PP rate in the DCB group was significantly higher than that in the POB group (48.0% vs 22.9%, *P* < 0.001; Table 3). The results of the K–M survival analysis using the log-rank test were similar (Figure 2). The HR obtained from the adjusted Cox regression analysis was 2.303 (95% CI: 1.518–3.495). However, the difference in TLR rates between the two groups was not statistically significant, with a 2-year cumulative rate of 25.0% for the DCB group vs 27.1% for the POB group (*P* ═ 0.767; Table 3). The survival curves were similar (Figure 3), and the adjusted HR was 0.956 (95% CI: 0.523–1.747). The sample size estimated using PASS software was 85 cases per group, and we achieved a sample size exceeding this estimate (100 cases in the DCB group and 140 cases in the POB group), indicating sufficient statistical power for the study.

**Table 3:** Outcome data after propensity score matching

**Outcome**	**DCB group**	***N***	**POB group**	***N***	***P* value**	**Hazard ratio with 95% confidence interval^†^**
PP (based on target lesion)	48 (48.0)	100	32 (22.9)	140	<0.001^*^	2.303 (1.518–3.495)
TLR (based on target lesion)	25 (25.0)	100	38 (27.1)	140	0.767	0.956 (0.523–1.747)
MA (based on limb)	5 (5.6)	89	5 (3.8)	132	0.529	43.284 (0.305–6144.367)
ACD (based on patient)	11 (14.5)	76	17 (14.3)	119	1.000	0.932 (0.293–2.962)
RC (based on limb)		77		115	0.292	–
0-3	30 (39.0)		33 (28.7)			
4	28 (36.4)		58 (50.4)			
5	10 (13.0)		13 (11.3)			
6	9 (11.7)		11 (9.6)			
ABI (based on limb)	0.50±0.18	72	0.43±0.16	110	0.005^*^	–

Observations are presented as “*n*(%)” or “± standard deviation”. ^*^Significant statistical difference due to a *P* value of less than 0.05; ^†^The value of hazard ratio (DCB vs POB) of maintaining PP or avoiding the other 3 outcomes. PP: Primary patency; TLR: Target lesion revascularization; MA: Major amputation; ACD: All-cause death; RC: Rutherford classification; ABI: Ankle brachial index; DCB: Drug-coated balloon; POB: Plain old balloon.

### Secondary outcomes

After matching, a new dataset No. 2 that met the common support assumption and parallel test assumption was obtained (Tables S3 and S4 and Figure S2). There were no significant differences in MA between the groups (Table 3 and Figure S3). Only after 3 variables (complete IPA before intervention, postoperative statin use, and postoperative antiplatelet use) were removed could multivariate Cox regression converge successfully. After a satisfactory dataset No. 3 was obtained, there were also no significant differences in ACD between the groups (Tables S5, S6, and 3 and Figures S4 and S5), without variable removal in the Cox regression analysis. In addition, there were no significant differences in the RCs at the end of follow-up between the groups (*P* ═ 0.292), but the ABI at the end of follow-up in the DCB group was significantly greater (mean 0.50 vs 0.43, *P* ═ 0.005) (Table 3).

## Discussion

This study retrospectively compared the 2-year outcomes of TASC C/D IPAD patients who underwent DCB and POB treatment. The results indicated that, compared to traditional POB, the application of DCB better maintained the PP in the lesions during the mid-term postoperative period (HR ═ 2.303, 95% CI: 1.518–3.495).

The results of other studies applying DCBs to IPA lesions are less consistent [17–22]. We believe this inconsistency may relate to the inclusion of more severe cases in studies that reported favorable outcomes for DCBs [21], [22]. In 2015, the TASC Steering Committee issued updated classification criteria for the severity of IPA lesions [5], which are widely adopted. We used this standard to screen for more severe patients with IPAD and retrospectively analyzed the superiority of DCBs over POBs. By employing the PSM method, we effectively reduced selection bias, enhancing the credibility of our analysis.

A recognized disadvantage of POBA is its high rate of restenosis and the subsequent need for TLR. Unlike coronary arteries of similar diameters, IPA lesions often involve longer segments and occur at multiple levels, leading to decreased flow rates and restenosis, even when immediate angiographic results are excellent [31]. The proliferation of SMCs is a significant cause of neo-intimal hyperplasia, ultimately resulting in restenosis [32]. The anti-proliferative mechanisms of drugs like paclitaxel and sirolimus help counteract SMC proliferation and reduce the restenosis rate, providing the theoretical basis for the widespread application of DCBs.

The first study on DCBs for IPAD was published in 2011 [17]. This prospective single-arm study utilized DCBs from IN.PACT™ (Medtronic Corp., Minneapolis, MN, United States) and reported a cumulative MA rate of 3.8% and a cumulative TLR rate of 16.3% at the 12th month. Since then, various related studies have emerged, most reporting early to mid-term results at the 6th or 12th month. A RCT, IN.PACT DEEP [33], and a retrospective cohort study [34] provided results at the 5-year mark, while only one study, a prospective single-arm investigation named BIOLUX P-III [35], reported 2-year results similar to those in the present study. Additionally, only one study specifically included TASC C/D IPAD patients [36], like ours, but reported results only at the 6th month.

Previous studies reported 1-year cumulative PP rates of 59%–73% after DCBA for IPADs [18], [33], [37]. Our present study, which included only severe lesions, achieved a 2-year cumulative PP rate of 48%, an encouraging outcome. The previously reported 1-year cumulative TLR rate after DCBA ranged from 8% to 30% [18], [20], [21], [33], [37], [38]. The 2-year cumulative TLR rate for severe lesions of 25% that we found appears acceptable. Furthermore, previously reported 1-year postoperative MA rates and ACD rates were 0%–16% and 2%–16%, respectively [17], [18], [20], [21], [33], [37–39], encompassing the 2-year rates we reported. In the BIOLUX P-III study, better rates of PP (83%) and TLR (9%) were reported compared to ours; however, the opposite was true for MA (26%) and ACD (21%). The loss to follow-up in this study reached 17% by the 6th month postoperatively, partly due to high mortality (7%). We believe this may explain the reported favorable PP and TLR rates.

The results of the present study demonstrated that for TASC C/D IPA lesions, the postoperative PP rate (*P* < 0.001) in the DCB group was superior to that in the POB group. This finding aligns with the 6-month results (*P* < 0.001) reported by two sub-studies [21], [38] from different countries in the AcoArt BTK trial, which used the same brand of DCBs as our study. This reflects the superiority of DCBs and indicates that significant restenosis of the target lesions may begin 6 months or even earlier after the procedure. However, we did not find a significantly lower postoperative TLR risk in the DCB group (*P* > 0.05). We believe this is due to many non-PP target lesions not yet causing clinical manifestations of CLI and therefore not undergoing revascularization. This finding is supported by the significant difference in the ABI between the groups at the end of follow-up (*P* ═ 0.005) and the equivalence of the RCs (*P* ═ 0.292) in the present study. This result is consistent with 1-year outcomes reported by some RCTs [20], [33]. Additionally, there was no difference (*P* > 0.05) in secondary outcomes, namely MA and ACD, between the groups, consistent with results reported by most RCTs [18], [20], [21], [33], [38]. Notably, the 95% CI of the HR value for MA in the DCB group was particularly broad (0.3–6144.4), which we attribute to a very low incidence of MA event count. Beyond PP, some unmeasurable factors may also influence these clinical outcomes, such as wound management practices and nursing experience. Their distributions may differ, potentially introducing some bias. Notably, the IN.PACT trial reported 5-year results that aligned with those of the present study [33]. This may indicate that restenosis of the target lesion tends to stabilize 1–2 years after balloon dilation angioplasty. In summary, we believe that DCBs have advantages over traditional POBs for TASC C/D lesions, particularly in maintaining postoperative PP. PP is the most direct indicator reflecting the severity of restenosis, which has historically been a challenge. Although there were no significant differences in other clinically relevant outcomes, milder restenosis correlated with better outcomes.

The present study has several limitations. First, it was a retrospective study, and despite the use of the PSM method, there is potential for selection bias. Second, it was conducted at a single center and utilized only one type of DCB, which limits the generalizability of the findings to the broader population. Third, we employed the TASC standard to screen for severe IPA lesions, which primarily assesses severity based on lesion length rather than the degree of calcification. The efficacy of DCBs is negatively correlated with the degree of calcification at the target lesion [40], [41]. We anticipate international, multicenter RCTs that incorporate multiple types of DCBs and address severe lesions from various perspectives.

## Conclusion

For severe IPA lesions classified as TASC C/D, DCBs are superior to POBs in maintaining PP for two years postoperatively, with no differences in other clinical outcomes such as TLR, MA, or ACD. Therefore, DCBs are a reliable treatment option.

## Supplemental data

Supplemental data are available at the following link: https://www.bjbms.org/ojs/index.php/bjbms/article/view/12157/3924.

**Graphical abstract**


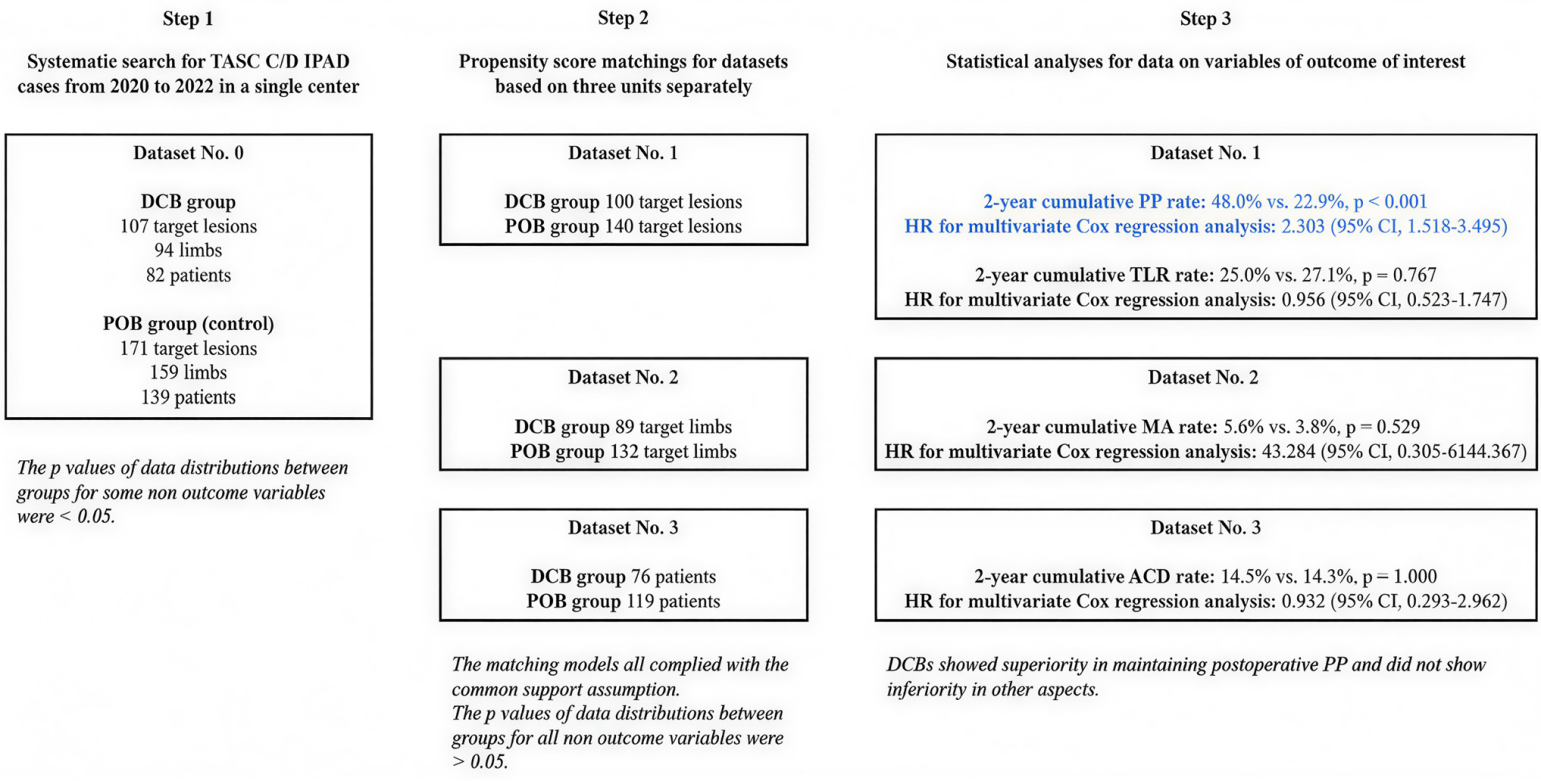


TASC: Trans-Atlantic Inter-Society Consensus; IPAD: Infrapopliteal arterial disease; DCB: Drug-coated balloon; POB: Plain old balloon; PP: Primary patency; TLR: Target lesion revascularization; MA: Major amputation; ACD: All-cause death; HR: Hazard ratio; CI: Confidence interval.
